# Age and Tumor Differentiation-Associated Gene Expression Based Analysis of Non-Familial Prostate Cancers

**DOI:** 10.3389/fonc.2020.584280

**Published:** 2021-01-26

**Authors:** Shashwat Sharad, Travis C. Allemang, Hua Li, Darryl Nousome, Anson Tai Ku, Nichelle C. Whitlock, Adam G. Sowalsky, Jennifer Cullen, Isabell A. Sesterhenn, David G. McLeod, Shiv Srivastava, Albert Dobi

**Affiliations:** ^1^Center for Prostate Disease Research, John P. Murtha Cancer Center Research Program, Department of Surgery, Uniformed Services University of the Health Sciences and Walter Reed National Military Medical Center, Bethesda, MD, United States; ^2^Henry M. Jackson Foundation for the Advancement of Military Medicine, Bethesda, MD, United States; ^3^Laboratory of Genitourinary Cancer Pathogenesis, National Cancer Institute, NIH, Bethesda, MD, United States; ^4^Pathology Center, Joint Pathology Center, Silver Spring, MD, United States

**Keywords:** age-associated gene expression, microarray, prostate cancer, tumor differentiation, laser captured microdissection, tumor-associate gene

## Abstract

Prostate cancer incidence in young men has increased. Patients diagnosed at an earlier age are likely to have aggressive prostate cancer and treatment decisions are continuing to be weighted by patient age and life expectancy. Identification of age-associated gene-expression signatures hold great potential to augment current and future treatment modalities. To investigate age-specific tumor associated gene signatures and their potential biomarkers for disease aggressiveness, this study was designed and stratified into well and poorly differentiated tumor types of young (42–58 years) and old (66–73 years) prostate cancer patients. The differentially expressed genes related to tumor-normal differences between non-familial prostate cancer patients were identified and several genes uniquely associated with the age and tumor differentiation are markedly polarized. Overexpressed genes known to be associated with somatic genomic alterations was predominantly found in young men, such as TMPRESS2-ERG and c-MYC. On the other hand, old men have mostly down-regulated gene expressions indicating the loss of protective genes and reduced cell mediated immunity indicated by decreased HLA-A and HLA-B expression. The normalization for the benign signatures between the age groups indicates a significant age and tumor dependent heterogeneity exists among the patients with a great potential for age-specific and tumor differentiation-based therapeutic stratification of prostate cancer.

## Introduction

Prostate cancer is known as a disease of old men and age is the greatest risk factor for cancer development. In the United States, the median age of diagnosis for men with prostate cancer is >75 years and only 10% of men young than 55 years are diagnosed with prostate cancer (1). However, the incidence of prostate cancers with poorly differentiated tumors is increasing in young men (2, 3). The prostate cancer associated mortality among young men with high grade tumor is much higher as compared to old men (4, 5). This suggests a distinct biology of prostate cancer development and the potential roles of unique oncogenic process between young and old men. Recently, it was shown that young prostate cancer patients had significantly higher inflammatory and immune responses to tumor development as compared to the old patients (6). Gene expression differences in early and late onset prostate cancer may influence early detection and treatment of prostate cancer. Prostate cancer incident rate and severity vary substantially by race, ethnicity, and geography. The understanding and identification of risk factors will assist in the development of more consistent screening parameters. It was also noted that men who develop prostate cancer before 50 years of age, are more likely to have a family history of prostate cancer. These men were also found to have worse clinicopathologic features, higher incidence of biochemical recurrence after radical prostatectomy, and lower survival probability (4, 7). Men who develop prostate cancer after 70 years of age had better clinicopathologic features, lower incidence of biochemical recurrence, and greater overall survival (8). These findings suggest a clinically relevant age-associated difference among men with prostate cancer.

Several other studies have linked prostate cancer to diet and altered metabolic conditions, such as obesity and diabetes. There is a contradictory report on young men with a family history of prostate cancer were less likely to have high-grade disease (9). To date, very few studies have focused specifically on aging and prostate cancer to better explain the genetic differences between young and old men with prostate cancer. Several disease-specific factors: tumor stage, tumor grade, prostate-specific antigen (PSA) level; and patient-specific factors: age, co-morbidity and functional status need to be considered in the decision-making process for the diagnosis and management of prostate cancer. To incorporate these important factors to select optimal treatment for individuals, several decision models have been published, yet their utility in clinical practice remains poorly understood. In general, prostate cancer is considered as a cancer of the elderly and the median age for prostate cancer diagnosis is around 66 years (between 65 and 74 years old). They men diagnosed before age 55 years were defined as early-onset prostate cancer. The recommendation of age specific prostate cancer management guidelines needs to be taken in account. There is a clear need to improve our understanding of the complex interrelationships between aging, tumor types, co-morbidities, and their impacts on expected outcomes. In this study, using laser capture microdissection of prostate cancer tumor cells and patient matched non-adjacent “non-malignant” prostate epithelial cells, we evaluated the genome-wide expression profiles in Caucasian men with no known family history of prostate cancer. The gene expression profiles were assessed in cells with well and poorly differentiated tumor cells morphology among old and young prostate cancer patients. for identification and validation of uniquely expressed genes. The goal of this study was to carefully identify and evaluate the comparative gene expression signatures from young and old prostate cancer patients stratified for similar clinicopathological features presented with tumor differentiation and recurring PSA (rPSA) at the time of radical prostatectomy.

## Materials and Methods

### Patient Cohort Selection and Study Design

**Figure 1:**
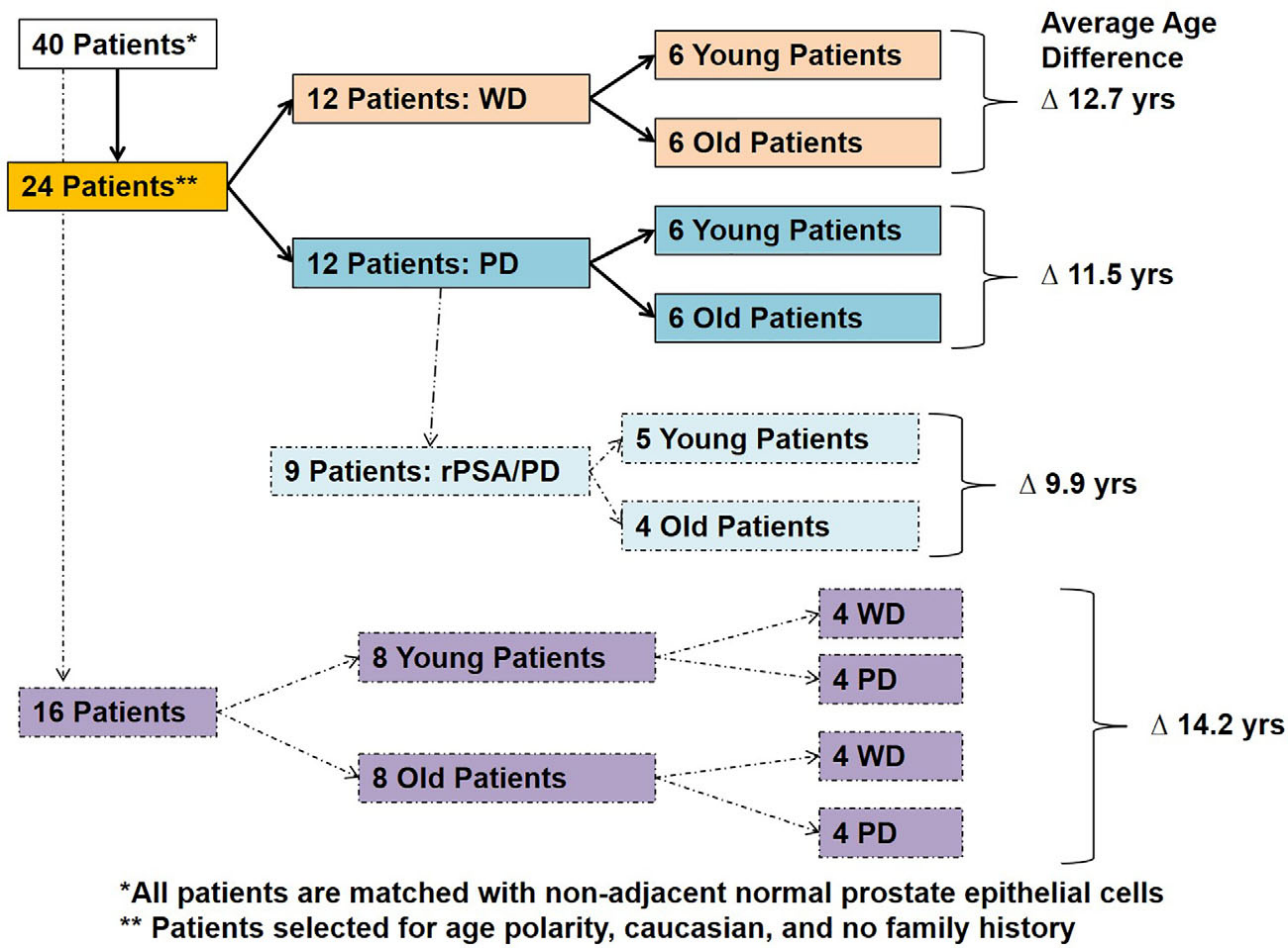
Patient selection and study design. Forty Caucasian American patients with well differentiated (WD) and poorly differentiated (PD) tumors with or without PSA recurrence (rPSA) were selected and divided into two groups based on age with an average age difference of 9.9–14.2 years; 1) Young and 2) Old.

The prostatic adenocarcinoma patients treated at the Walter Reed National Military Medical Center (WRNMMC) were enrolled at the Center for Prostate Disease Research (CPDR) from 1997 to 2010 under institutional review board approved protocol of WRNMMC 20405 and Uniformed Services University of the Health Sciences (USUHS) 20311. Prostate tissue specimens and clinical data used in this study were obtained under above IRB-approved protocol and informed consent was obtained from each subject. Prostate tumor samples and adjacent histologically normal tissues were obtained from patients that underwent radical prostatectomy (RP). The tissue sections were frozen and stored in optimal cutting temperature (OCT) compound at -80^0^C. Over 300 radical prostatectomy tumor and adjacent benign specimens of a PSA-screened patient with no prior androgen ablation treatment were evaluated and eligible for selection into the study. Forty unique patients met the inclusion criteria of race (Caucasian American), age (young and old), and tumor differentiation (well and poorly) from the initial cohort. Well differentiated tumor cells were obtained from specimens with Gleason sum 6–7 with no seminal vesicle invasion and with no PSA recurrence (rPSA) and poorly differentiated tumors were defined with a Gleason sum 8-9 with PSA recurrence in 65% of cases. PSA recurrence was defined as two consecutive times of PSA > 0.2 ng/ml with follow up from surgery. Laser capture microdissection (LCM) was performed on 80 specimens from 40 patients and were sub grouped based on the age and tumor differentiation (**Table 1** and **Figure 1**). The criteria for the inclusion of “young (42–58 years)” and “old (66–73 years)” patients with minimum average age difference of at least ~10 years (~9.9 years to ~14.2 years) were normalized for natural aging related gene signatures and also to define the true young (≤ age 58) and old (≥ age 66) age in the context of prostate cancer (**Table 1** and **Figure 1**). Also, this study is a longitudinal cohort of military health-care beneficiaries and this setting reduces disparity in socioeconomic status, health-care access, and lifestyle factors that potentially influence prostate cancer progression.

**Table 1:** Patient selection clinical data.

	Tumor Differentiation			
Patient Group	Well Differentiated “WD”	Poorly Differentiated “PD”	Balanced (WD & PD)	Recurrent PSA
**Number (n)**	12	12	16	09
**Age Range [Young], [Old]**	[Young], [Old]	[Young], [Old]	[Young], [Old]	[Young], [Old]
	(n=6), (n=6)	(n=6), (n=6)	(n=8), (n=8)	(n=5), (n=4)
	[42–58], [66–73]	[50–62], [68–72]	[42–59], [65–73]	[56–63], [68–70]
**Average Age Difference**	12.7 years	11.5 years	14.2 years	9.9 years
**Family History**	None	None	None	None

“WD”, well differentiated; “PD”, poorly differentiated; “Balanced”, balanced differentiation; “rPSA”, recurrent PSA.

### Laser Capture Microdissection and RNA Extraction

The selection of both the benign prostate epithelial cells with normal morphological appearance (N) and prostate tumor epithelial cells (T) from hematoxylin and eosin stained frozen tissue sections was performed by using the PixCell II Laser Capture Microdissection System (LCM, Arcturus, Mountain View, CA, USA). Approximately 5000 cells from morphologically normal fields of nonadjacent prostate epithelial cells and poorly/well differentiated morphology were captured and collected from tumor foci. All captured normal benign and tumor epithelial cells were further processed for RNA extraction by using Arcturus Paradis RNA extraction and isolation kit. The isolated RNA was quantified by using RiboGreen dye (Molecular Probes, Eugene, OR, USA) and Versa-Fluor fluorimeter (BioRad, Hercules, CA, USA).

### RNA Labeling, Hybridization, and Gene Expression

The linear amplification of the RNA was done by using the Arcturus Paradise RNA amplification kit as per the manufactures protocol. Two nanograms of total RNA was used for the cDNA synthesis and biotinylation steps. The biotinylation of poly (A) RNA was carried out by using MEGA script T7 *in vitro* Transcription Kit (Ambion, Austin, TX, USA). After biotinylation step the RNA was further purified by QIAGEN RNeasy spin columns (Qiagen, Germantown, MD, USA) as per manufacturer’s protocol. Linearly amplified biotin labeled RNA samples were hybridized to a high-density oligonucleotide human genome array HG-U133A Affymetrix GeneChip Arrays at 42°C for 16 h and prepared according to previously described methods (10, 11). The hybridized GeneChip arrays were washed, stained and scanned with the HP GeneArray Scanner (Hewlett-Packard, Santa Clara, CA, USA) controlled by GeneChip 3.1 Software (Affymetrix, Thermo Fisher Scientific, Waltham, MA, USA).

### GeneChip Expression Data Analysis

**Figure 2:**
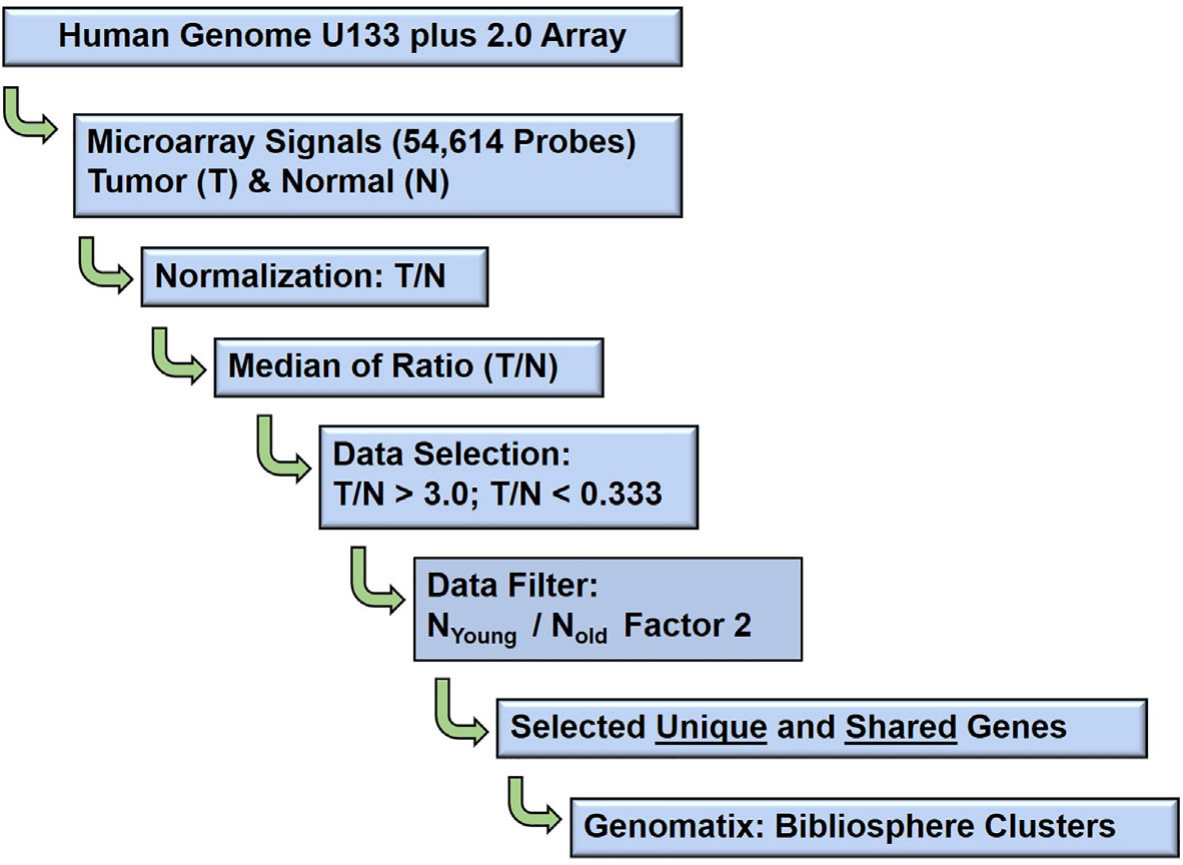
Schematic diagram: Microarray data analysis workflow. The bioinformatics data analysis of age (young and old) associated gene expression responses in well, poorly, balanced differentiated tumors and biochemical (PSA) recurrence in prostate cancer patients.

Schematic bioinformatic data analysis workflow of the raw gene expression data output (CEL files) of 80 GeneChip analysis (HG U133A array, Affymetrix, Santa Clara, CA, USA) are presented in **Figure 2**. The probe intensity of Microarray GeneChip images were captured and analyzed by Affymetrix GeneChip^®^ Microarray Analysis Software, version 3.1 and Affymetrix Micro DB and Data Mining Tool version 2.0 (Affymetrix, Thermo Fisher Scientific, Waltham, MA, USA), and Statistica version 4.1 (Stat Soft, Inc., Tulsa, OK, USA). Further, the CEL files of raw gene expression data were processed by statistical computing language R (Bioconductor package). The background subtraction and normalization were done by Robust Multi-array Analysis (RMA, http://rmaexpress.bmbolstad.com) and by the ChipInspector a single-probe analysis approach (Genomatix GmbH, Munich, Germany; http://www.genomatix.de). To improve the signal-to noise ratio, increase the statistical stringency, and to eliminate probe mismatches or multiple matches, the single probes matching to the transcripts and normalization of total intensities was performed by the Significance Analysis of Microarrays (SAMs) and enrichment of significantly altered signal intensities approach (12). The signal of probe intensities which met both RMA and ChipInspector normalization criteria with a false discovery rate of p 0.05% yielded significantly up and down regulated probes. The signal intensities below 30 were excluded from both the tumor and corresponding normal probe for further analyses. The normalized data were then used to calculate the fold changes dividing gene expression signal value of Tumor over Normal (T/N), and then applying 2, 2.5 (data not shown) and 3 cut-offs. Probes were then matched to genes. In this study high stringent criteria were used and the genes with fold change T/N > 3 and T/N < 0.33 were differentially expressed as up or down regulated genes. The Genomatix-GePS and DAVID (NAÏVE-DAVID) software were used for the functional gene ontology and venn diagram analysis (13). The gene network analysis for the selected genes was performed by using Genomatix pathway edition of Bibliosphere (Genomatix GmbH, Munich, Germany, www.genomatix.de) as previously described methods (12–14). The network and pathway analysis as previously described methods (15).

### Gene Ontology and Pathway

The unique age associated genes of young and old prostate cancer patients in well differentiated, poorly differentiated, balanced differentiated and recurred prostate-specific antigen in poorly differentiated tumors (rPSA-PD) were queried into the Genomatix Pathway System (GePS) which utilize the expert-curated GO information from public and proprietary databases (Genomatix GmbH). Independently, these age and tumor type associated genes were also queried by the Database for Annotation, Visualization and Integrated Discovery (DAVID) software (http://david.abcc.ncifcrf.gov) (13). The DAVID software runs the clustering algorithm to classify highly related genes into functionally related groups used to reveal the tight association of genes associated with age, tumor type and rPSA. The gene IDs of all the differentially expressed genes with their corresponding fold change values of the studied group of young and old prostate cancer patients were entered the BiblioSphere knowledge-based pathway analysis software (Genomatix GmbH) for functional network, canonical pathway and gene ontology analysis (10). The functional Classification Tool was utilized to evaluate the functional similarity between queried input genes (10, 16). The software generates the interaction between genes and connects by co-citation within one sentence at abstract levels. The significance of enriched genes mapped to different canonical pathways was calculated by the Fischer’s exact test (p-value). The color code in the network is related to fold changes (red indicating up-regulation and blue downregulation). Genes with the highest number of interactions forming the central node in the network were considered as most significant and were further analyzed to evaluate significant probe-signal intensities individually in tumor and benign samples. Canonical pathways and gene ontology terms were ranked by log (p-value) (10, 16).

### RNA Seq Expression Data Analysis From the Publicly Available Human Gene Samples

Publicly available RNA-Seq from the Cancer Genome Atlas (TCGA) and associated clinical processed data were downloaded from the recount2 project for 319 cases (https://jhubiostatistics.shinyapps.io/recount/) of Caucasian men. Only cases matching primary tumors and within the age threshold (42–58 and 66–73) were retained for analysis. The DESeq2 R/Bioconductor package was used to read and perform analysis of the RNA-seq count data. Differential expression tests were used to compare the differences between young and old patients adjusting for Gleason score (≤3 + 4 and ≥4 + 3). Unsupervised hierarchical clustering was used after a variance stabilizing transformation of the raw count data.

Furthermore, 46 pairs of paired-end RNA-Seq data from TCGA(https://gdc.cancer.gov) corresponding to prostate tumor (n=23) and matching normal tissue (n=23) from 23 patients were selected based on race (Caucasian) and age (young: 42–58, old: 66–73). Briefly, reads were filtered by quality and complexity prior to alignment to human reference genome (hg19) using Star and aggregated by featureCounts. Genes with less than five counts in at least 50% of the samples were filtered out. Differential expression analysis between tumor and normal samples was performed using DESeq2 after stratifying the dataset by age (young and old) and Gleason score (well differentiated: Gleason score ≤3 + 4, poorly differentiated: Gleason score ≥4 + 3). Differentially expressed genes were filtered by false-discovery rate of 0.05 and a log2 fold change less than -1 or greater than 1.

## Results

### Selection of Young and Old Gene Patients

In this experiment, we evaluated tumor samples from 40 Caucasian American (CA) prostate cancer patients who underwent radical prostatectomy from a common and homogenous tumor subtype, and recurrent PSA from 40 Caucasian American (CA) prostate cancer patients. The old and young prostate cancer patients were selected based on their age, race, cellular differentiation status, and by no indication of family history of prostate cancer (**Table 1**). The patient age referenced is the age at which the patient underwent radical prostatectomy surgery. In this study, a patient was said to have no family history if he did not have any known first or second-degree relatives with a history of prostate cancer. None of these patients underwent neoadjuvant chemotherapy or radiation therapy prior to date of prostatectomy. The Laser Captured Microdissection (LCM)-selected individual tumor and normal cells from RP specimens were matched by the histological cellular differentiation status. The Gleason score of the patients within the Well Differentiated “WD” group was equal to 7 (≤3 + 4) or less, whereas the Gleason score of the patients in the Poorly Differentiated “PD” group was equal to 7 (≥4 + 3) or greater. The 16 patients within the balanced differentiation “Balanced” subset and the nine patients within the recurrent prostate specific antigen “rPSA” subset were selected from the 24 patients which make up the two primary groups WD and PD (**Figure 1**). The average age difference between young and old prostate cancer patients’ groups were 12.7 years (Well Differentiated “WD”), 11.5 years (Poorly Differentiated “PD”), 14.2 years (Balanced-WD & PD), and 9.9 years (Recurrent PSA “rPSA”). The tumor and matching histologically normal prostate epithelial cells from each specimen, were isolated and total RNA were extracted to measure the gene expression levels by microarray analysis (10, 17–19). The clinical, histopathological and demographic characteristics of the study population stratified by age and differentiation are summarized in **Supplementary Table 1**; PD (1A), WD (1B), Balanced (1C) and rPSA (1D).

### Identification of Differentially Expressed Prostate Cancer Gene Signature of Young and Old Gene Patients

**Figure 3:**
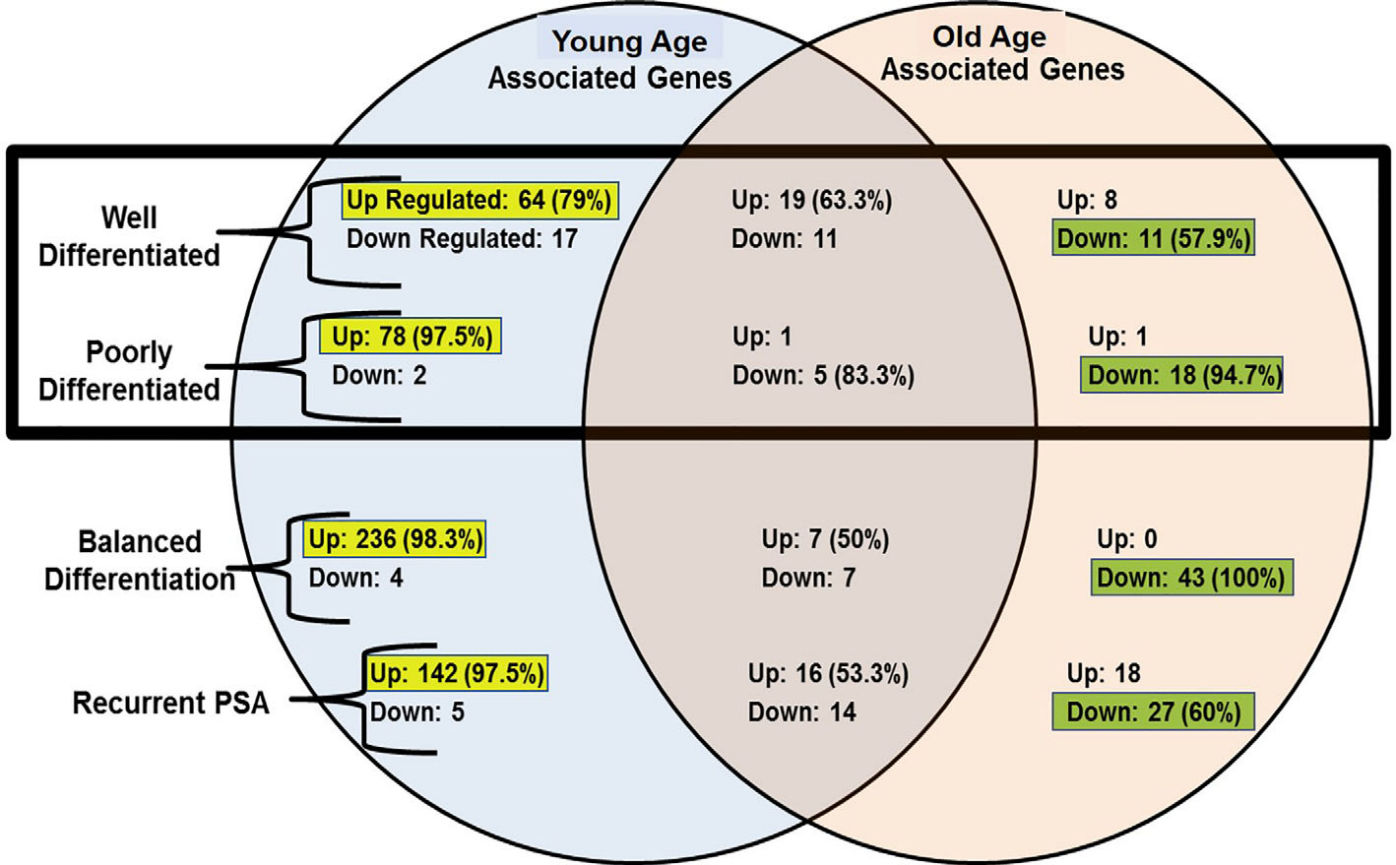
Venn-diagram summary of gene expression results; “Up”, Up-Regulated Gene Expression; “Down”, Down-Regulated Gene Expression; “-Associated Genes” Genes with expression levels that surpass the factor 3 inclusion criteria. The up-regulated genes are more common in young prostate cancer patients whereas old patient’s gene profile carry mostly down-regulated genes.

**Figure 4:**
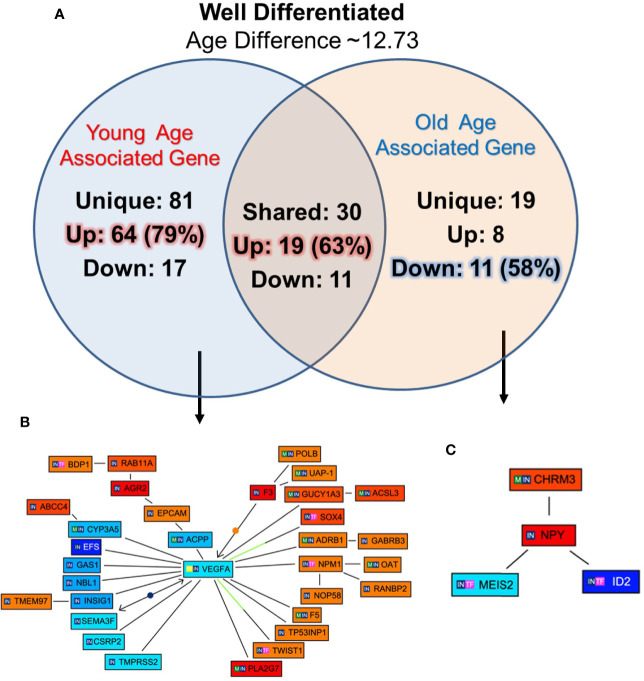
Functional analysis of differentially expressed genes in well differentiated tumors of young and old prostate cancer patients by GePS system. **(A)** The total 81 genes were uniquely associated with young age [64 (79%) genes up-regulated and 17 (21%) genes down-regulated] and 19 genes with old age [8 (42%) up-regulated and 11 (58%) down-regulated]. There are 30 genes [19 (63%) up-regulated and 11 (37%) down-regulated were common between the both the groups. **(B, C)** The Venn diagram analysis represents significant genes with at least 3-fold expression. The hierarchical cluster showing the expression levels of the 32 significant genes associated with WD-young group **(B)** and 04 genes with WD-old group **(C)**. The unique genes associated with WD young and old group were imported into GePS. Orange/red color shows up-regulation and blue color shows down-regulation. The intensity of blue and red colors indicates the degree of up or down-regulation, respectively. A solid line represents an expert curated association between the two gene products and a dotted line means there is an association by co-citation. Expert level filter settings were used to generate the network, which contains interactions curated by experts (Genomatix and NetPro) based on literature and genes without any interactions were filtered out.

The gene expression features were normalized using the RMAExpress and ChipInspector software’s. The gene expression signals were calculated using their patient patched tumor over normal (T/N) expression ratio and the median signal values calculated. A stringent factor of 3X cut off was applied to enrich gene expression signatures to determine the significantly expressed genes. Young and old age group unique gene expression features were further analyzed based on their tumor histological differentiation and rPSA. The tumor signature was also normalized for the benign signature to minimize the normal aging caused differences. A Venn-Diagram was performed to evaluate the shared and unique signatures among the groups, WD, PD, Balanced Differentiation, and rPSA. The unique gene expression features of the young age group were matched to 520 up-regulated and 28 down-regulated genes. Of these gene expressions unique to young men, the majority of unique gene expressions were found to be upregulated within the WD, PD, balanced and rPSA groups respectively, 79% (64 genes), 97.5% (78 genes), 98.3% (236 genes), and 97.5% (142 genes). In the old group, 27 genes were up-regulated and 99 genes down-regulated, respectively (**Figure 4**). Of these gene expressions unique to old men, most unique gene expressions were found to be downregulated within the WD, PD, balanced and rPSA groups respectively, 57.9% (11 genes), 94.7% (18 genes), 100% (43 genes), and 60% (27 genes). These results suggest the existence of strong age-tumor associated difference in gene expression profile. Young men with prostate cancer tend to have more up-regulated genes whereas in the old men are mostly down regulated (**Figure 3**).

### Gene Signature Unique to Well-Differentiated Tumors of Young and Old Prostate Cancer Patients

To identify unique pathway/network of genes associated with WD tumor in old/young prostate cancer patients, 12 WD (six old and six young) patients were identified with 12.73 years age difference. The mean age for the WD-Young and WD-old patients were ~53.9 and ~66.6 years respectively. All the differentially expressed genes for both the groups were queried for Genomatix Network and Pathway Analysis (GePS). The 81 genes were uniquely expressed in young patient’s-WD tumor and 19 genes in Old-WD tumors. Interestingly, 79% (64 genes) were up regulated in young-WD tumor and 58% (11 genes) were down regulated in old-WD tumor (**Figure 4A**). To further evaluate the impacted signaling pathways, we constructed the pathway/network system of all the differentially expressed genes unique to old/young-WD tumors with cut-off over 3-fold change (**Figures 4B, C****)**. This analysis revealed Vascular Endothelial Growth Factor A (VEGFA), which is down regulated in young patients with well differentiated tumors as a central node based on the gene score (score represents numerous interactions of a gene). Further, Neuropeptide Y (NPY) gene was found to be upregulated in old patients with well differentiated tumors as a central node. A list of all the unique and shared gene for the well differentiated (WD) are tabulated in **Supplementary Figures 1A–C**.

### Gene Signature Unique to Poorly Differentiated Tumors of Young and Old Prostate Cancer Patients

**Figure 5:**
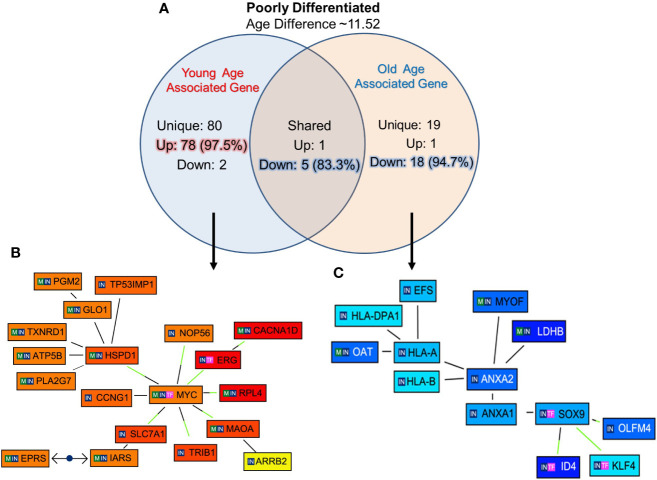
Functional analysis of uniquely expressed genes in poorly differentiated tumors of young and old prostate cancer patients by GePS system. **(A)** The total 80 genes were uniquely associated with young age [78 (97.5%) genes up-regulated and 02 (2.5%) genes down-regulated] and 19 genes with old age [01 (2.3%) up-regulated and 18 (94.7%) down-regulated]. There are six genes [01 (16.7%) up-regulated and 05 (83.3%) down-regulated were common between the both the groups. **(B, C)** The Venn diagram analysis represents significant genes with at least 3-fold expression. The hierarchical cluster showing the expression levels of the significant genes associated with Poorly Differentiated (PD)-young group **(B)** and PD-old group **(C)**. The unique genes associated with PD young and old group were imported into GePS. Orange/red color shows up-regulation and blue color shows down-regulation. The intensity of blue and red colors indicates the degree of up or down-regulation, respectively. A solid line represents an expert curated association between the two gene products and a dotted line means there is an association by co-citation. Expert level filter settings were used to generate the network, which contains interactions curated by experts (Genomatix and NetPro) based on literature and genes without any interactions were filtered out.

The unique genes associated with PD tumor of old and young prostate cancer patients, 12 PD (six old and six young) prostate cancer patients were identified with 11.52 years age difference. The mean age for the PD-Young and PD-old patients were ~57.2 and ~68.8 years respectively. All the differentially expressed genes for the age groups were queried for Genomatix Network and Pathway Analysis (GePS). It was found that 80 genes were uniquely expressed in young-PD tumor cohort and 19 genes in old-PD tumor cohort. Remarkably, 97.5% (78 genes) were up-regulated in young-PD tumor and 94.7% (18 genes) were down-regulated in old-PD tumor (**Figure 5A**). To further understand the significantly altered signaling pathways, the pathway/network were constructed for all the differentially expressed genes unique to old/young-PD tumors with cut-off of 3-fold change (**Figures 5B, C****)**. This analysis revealed the MYC Proto-Oncogene, BHLH Transcription Factor (*MYC*) and ETS Transcription Factor ERG (*ERG*) were upregulated in young patients with poorly differentiated tumors as the central node based on the gene score (score represents numerous interactions of a gene). Further, Annexin A2 (*ANXA2*) gene was found to be down-regulated in old patients with poorly differentiated tumors as a central node, and inhibitor of Differentiation (*ID4*), human leukocyte antigens (*HLA)-A/B* were down regulated in old patients. A list of all the unique and shared genes for the poorly differentiated (PD) group are tabulated in **Supplementary Figures 2A–C**.

### Gene Signature Unique to Balanced Diffrentited Tumors of Young and Old Prostate Cancer Patients

**Figure 6:**
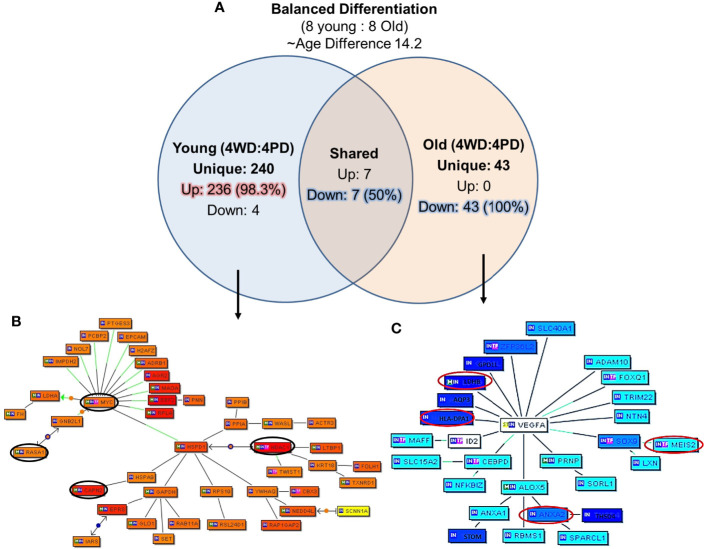
Uniquely expressed genes in balanced differentiated tumors of young and old prostate cancer patients. **(A)** 240 genes were uniquely associated with young age and 98.3% genes are upregulated. All uniquely expressed genes among the old patients were down regulated. Marked age-associated differences in gene expression signatures have been identified in prostate tumors **(B)** *MYC/HDAC1/RASA1*, *CAPN2* genes were elevated in young patients, and **(C)** *VEGFA/MEIS2/HLA/LDHB/ANXA2* genes were down-regulated in old patients. The Venn diagram analysis represents significant genes with at least 3-fold expression. The hierarchical cluster showing the expression levels of the significant genes. Orange/red color shows up-regulation and blue color shows down-regulation. The intensity of blue and red colors indicates the degree of up or down-regulation, respectively. A solid line represents an expert curated association between the two gene products and a dotted line means there is an association by co-citation. Expert level filter settings were used to generate the network, which contains interactions curated by experts (Genomatix and NetPro) based on literature and genes without any interactions were filtered out.

The unique genes and their network associated with balanced differentiation (WD and PD) tumor in young and old prostate cancer patients, eight young and eight old prostate cancer patients were identified with WD and PD tumor (four young and four old men in each group) patients were identified The mean age difference between young and old group was 14.2 years. The uniquely expressed genes for the groups were queried with Genomatix Network and Pathway Analysis (GePS). The 240 genes were uniquely expressed in young group and 43 genes in old group. Interestingly, all the 43 (100%) genes were down-regulated in old group, whereas 98.3% (236 genes) were up-regulated in young prostate cancer patients (**Figure 6A**). Among the genes shared among the young and old patients, seven (50%) were upregulated and seven (50%) were down regulated. Further signaling pathways/network were constructed of all the uniquely expressed genes with cut-off over 3-fold change (**Figures 6B, C****)**. *MYC* Proto-Oncogene, *HDAC1* (Histone Deacetylase 1) and *HSPD1* [Heat Shock Protein Family D (*Hsp60*) Member 1] were upregulated in young patients forming central node with several gene. The *RASA1* (RAS P21 Protein Activator 1) inhibitory regulator of the Ras-cyclic AMP pathway and suppressor of RAS function and CAPN2 (*Calpain 2*), muscle-specific proteins were also found to be up-regulated. Among the old patients, *VEGFA* (Vascular Endothelial Growth Factor A) was found to be down regulated forming a central node. The *HLA* (human leukocyte antigen), *LDHB* (Lactate Dehydrogenase B), lipocortin I (Annexin A1, *ANAX1*), *ANAX2* (Annexin A2), *MEIS1* (Meis Homeobox 1), developmental genes such as *SLC40A1* (Solute Carrier Family 40 Member 1), *FOXQ1* (Forkhead Box Q1), DNA binding protein *SOX9* [SRY- Sex Determining Region Y)-Box 9], *ID2* (Inhibitor Of DNA Binding 2), immune system and developmental biology related gene, *CEBPD* (CCAAT Enhancer Binding Protein Delta), and *LDHB* (Lactate Dehydrogenase B) previously described as hypermethylated in prostate cancer were down-regulated in old prostate cancer patients.

### Gene Signature Unique to Biochemical Recurrence and Age

**Figure 7:**
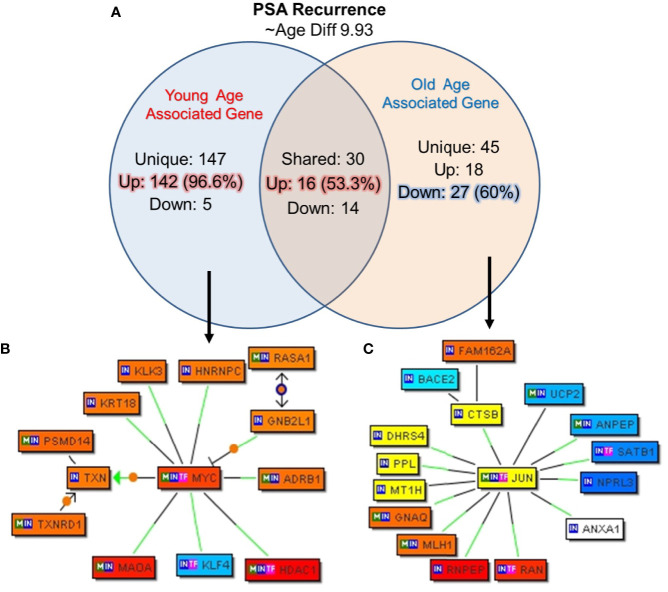
Uniquely expressed genes in PSA recurrence group of young and old prostate cancer patients. **(A)** 147 genes were uniquely associated and 96.6% (142) genes were up-regulated in rPSA-young prostate cancer group. In old group 45 genes were uniquely expressed and 60% (27) genes were down regulated. Thirty genes were found to be co-expressed in both the groups. ~53% (N=16) were up-regulated and ~47% (N=14) genes were down-regulated. rPSA and marked age-associated differences in gene expression signatures were identified in prostate tumors **(B)** *MYC* form a central node with *HDAC1, RASA1*, and *KLK3*/PSA genes and were down-regulated in young-rPSA patients, and **(C)** *JUN* form a central node with *ANAX1, NPRL3, SATB1, UCP2, DHRS4, CTSB, PPL, BACE*2, and *MLH1* in old-rPSA patients. The Venn diagram analysis represents significant genes with at least 3-fold expression. The hierarchical cluster showing the expression levels of the significant genes. Orange/red color shows up-regulation and blue color shows down-regulation. The intensity of green and red colors indicates the degree of up or down-regulation, respectively. A solid line represents an expert curated association between the two gene products and a dotted line means there is an association by co-citation. Expert level filter settings were used to generate the network, which contains interactions curated by experts (Genomatix and NetPro) based on literature and genes without any interactions were filtered out.

In this cohort of prostate cancer patients 22.5% (nine out of 40) patients reported PSA recurrence after a follow-up, we further analyzed the unique genes associated with biochemical recurrence (rPSA) and age by creating subgroup of the five young patients and the 4 old patients with rPSA. The mean age difference between the young and old patients was 9.93 years. The uniquely expressed genes for both groups were queried for Genomatix Network and Pathway Analysis (GePS). Among the young men with rPSA, 147 genes were uniquely expressed with 142 genes (96.5%) up-regulated. Among the old men with rPSA, 45 genes (60%) were down-regulated (**Figure 7A**). The uniquely expressed genes that were common between the young and old men with rPSA were found to have nearly equal proportion upregulated (53%) vs down regulated (47%).

Further signaling pathways/network were constructed of all the uniquely expressed genes with cut-off over 3-fold change (**Figures 7B, C**). In young-rPSA group, *MYC* Proto-Oncogene and in old-rPSA group *JUN* (Jun Proto-Oncogene, *AP-1* Transcription Factor Subunit), *Wnt* signaling pathway associated gene were populated as central node. The gene network analysis showed *HDAC1* (Histone Deacetylase 1), *RASA1* (RAS P21 Protein Activator 1), and Prostatitis and urethral stricture associated and coregulator of androgen receptor activity *KLK3/*(PSA) (Kallikrein Related Peptidase 3) gene tightly associated with *MYC* transcription factor in young-rPSA group. On the other hand in old-rPSA group he network of genes such as lipocortin I (Annexin A1, *ANAX1*), mTOR signaling pathway associated gene *NPRL3* (NPR3 Like, GATOR1 Complex Subunit), bladder urothelial carcinoma and Breast disease associated gene *SATB1* (SATB Homeobox 1), body mass index and metabolism associated gene *UCP2* (Uncoupling Protein 2) and *DHRS4* (Dehydrogenase/Reductase 4), NOD-like receptor signaling pathway associated gene *CTSB* (Cathepsin B), developmental biology and butyrophilin (*BTN*) family interactions associated gene *PPL* (Periplakin), Down Syndrome and Alzheimer Disease associated gene *BACE2* (Beta-Secretase 2) and hereditary, colorectal cancer, mismatch repair cancer syndrome associated TP53 activity regulator *MLH1* (MutL Homolog 1) gene was found to be associated with JUN transcription factor.

### Validation of Gene Signature Unique to Poorly and Well Differentiated Tumors of Young and Old Prostate Cancer Patients in The Cancer Genome Atlas Database

**Figure 8:**
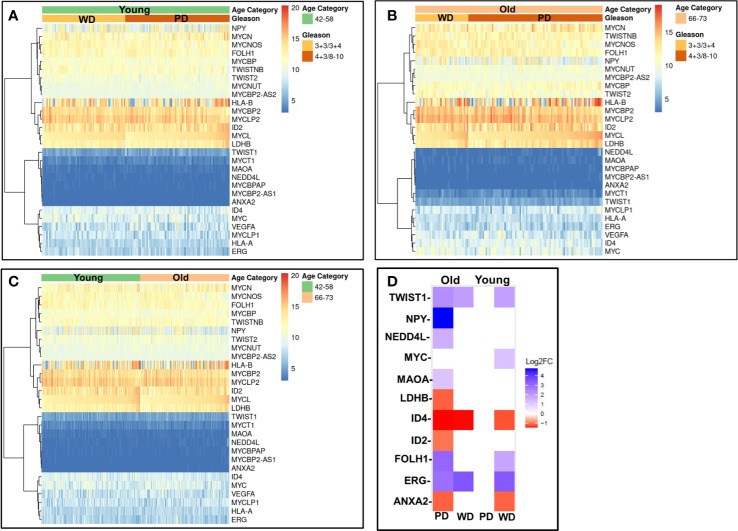
Validation of gene expression signatures. Gene profile of old/young age-associated well/poorly differentiated prostate tumors were cross-referenced using RNA-seq gene expression data from recount2 project **(A–C)** and prostate cancer TCGA cohort **(D)**. The differentially expressed genes (DEGs) of interest were investigated for age (young and old) and Gleason/Differentiation (Well (and Poorly). **(A)** The gene of interest signature of young poorly differentiated (PD) and well differentiated (WD) tumors; **(B)** old PD and WD tumors, and **(C)** the young (42–58 years) and old (66–73 years) are presented in heatmap form. **(D)** DEGs of interest are presented in the table and heatmap.

To validate our findings, we first analyzed RNA-seq gene expression along with clinical data from the recount2 project for 319 cases Caucasian men. The matching primary tumors within the age threshold were analyzed (**Figure 8**) for age (young and old) and Gleason score ≤3 + 4 (WD) and ≥4 + 3 (PD). The gene signature profile of young PD and WD tumors (**Figure 8A**), old PD and WD tumors (**Figure 8B**) and gene signatures for the young (42–58 years) and old (66–73 years) (**Figure 8C**) were consistent with our discovery cohort. All the up and down regulated gene panel of WD-old/young and PD-old/young present in our initial findings are validated in this cohort.

To further confirm these findings, we accessed 46 samples from the prostate TCGA cohort consisting of prostate tumor (n=23) and matching normal tissue (n = 23) from 23 patients were stratified by age (young: 42-58, old: 66-73), Gleason score (well differentiated: Gleason score ≤3 + 4, poorly differentiated: Gleason score ≥4 + 3) and race (Caucasian American). Clinicopathological features and clinical data such as patient ID, race, family history, Gleason score, PSA at diagnosis, clinical stage, pathological stage, and biochemical recurrence for the old WD, young WD, old PD, and young PD used for the TCGA analysis were summarized in the **Supplementary Tables 2A, B**. The differentially expressed genes (DEGs) of interest were validated and presented in the table and heatmap (present/absent and up/down regulated) (**Figure 8D**). The *ERG/MYC* was up and *ANXA2* was down regulated in Young-WD whereas *FOLH1/PSMA* was up regulated in both young-WD and old-PD group. The genes *NPY/NEDD4L/MAOA/TWIST1* were up regulated and ID4 down regulated in old-PD group. The gene *LDHB* was down regulated in both WD-young and PD-old group of patients (**Figure 8D**).

## Discussion

Prostate cancer is an age-associated disease and behaves very heterogeneously in clinical aggressiveness. An increased incidence of prostate cancer in young men has been reported. The biological difference of prostate cancer development between young and old men is not clearly understood. This study describes the results of transcriptome profiling of clinically localized and lymph node-negative prostate cancer. Through gene expression profiling, we investigated the men diagnosed with prostate cancer and treated with radical prostatectomy at young and old ages with well and poorly differentiated tumors, as well as those that developed PSA recurrence. The individual cellular differentiation classified as well differentiated (WD) or poorly differentiated (PD) was determined histologically. Several genes have been identified that are potentially associated with the unfavorable prognosis of the tumors. We found that most of the unique genes expressed in young patients are upregulated in in all the tumor types and rPSA group, whereas in old patients all the uniquely expressed genes were predominantly down regulated, suggesting the fundamental tumor development biology differences associated with patient age. This tumor and age associated gene expression difference was seen across all the subgroups that we evaluated in young/old men with WD/PD/Balanced and rPSA. In well differentiated tumor type the up regulation of *VEGFA* was identified as a key central node in young prostate cancer patients and down regulation of *NPY* in old men. *VEGF* is a sub-family of growth factors and the key mediator of angiogenesis in cancer for cancer development and growth. NPY is a secretary plasma protein mostly over expressed in prostate cancers. The upregulation of *VEGF* and down regulation of *NPY* can serve as potential prognostic and diagnostic markers for the well differentiated tumor type of young and old men.

*c-MYC* is often upregulated leading to increased expression of several genes involved in cell proliferation and cancer development such as carcinoma of the cervix, colon, breast, lung, and stomach (20). In this study, we found the up regulation of *MYC* proto oncogene in only poorly differentiated tumor of young prostate cancer men. Interestingly, instead of *MYC*, calcium-dependent phospholipid-binding protein *ANXA2* was downregulated in PD tumor of old men forming a key central node. The main function of ANXA2 is to establish exocytosis of intracellular proteins to the extracellular domain and interferes with various cellular processes. In cancers, *ANXA2* plays a role in disease progression and down regulation of mRNA expression correlates with resistance to treatment, binding to the bone marrow, histological grade and type, TNM-stage and shortened overall survival. The regulation of Annexin A2 (*ANXA2*) is one of the potential targets for cancer management and treatment (21). In prostate cancer, *ANXA2* module inversely correlated with ERG in its network and can be used for biological stratification and therapeutic targeting of ERG based stratification of prostate cancers (22). Immunotherapy works by activating the patient’s own immune system to fight cancer. Human leukocyte antigen class I (HLA-I) molecules are important for effective tumor killing. CD8+ T cells recognize tumor peptides presented by major *HLA-I* genes (*HLA-A*, *HLA-B*, and *HLA-C*). Here we found that HLA-A/B decreases in old patients. The down regulation of *HLA-A/B* genotypes can influence the responsiveness of the old men to immunotherapy (23). Down-regulation of immune-related pathways, and especially the pathway involved in immuno-suppression, may be a common mechanism related to prostate cancer onset in old men.

In the balanced differentiated group of young and old prostate cancer patients, *MYC and HDAC1* genes were emerged as a central node and uniquely upregulated in young men whereas *VEGFA is* the key node in old men and found to be down regulated along with *HLA* and *ANXA2* genes in old patients. This set of genes can also serve as the potential age differentiated marker for prostate cancer development and progression in old and young men.

We have also evaluated PSA recurrence in old and young men as disease progression parameter. Our results propose new age specific gene signatures unique to biochemical recurrence of prostate cancer. Interestingly, both the young and old prostate cancer men with PSA recurrence bear poorly differentiated tumor only that could be due to the small sample size. The up-regulation of proto-oncogene *MYC* and down-regulation of *JUN* transcription factor have been populated as central node forming gene in young and old men with PD tumor respectively with PSA recurrence (24).

The genes identified as central regulatory nodes were *ANXA2* which was down-regulated in old patients with poorly differentiated tumor type, *VEGFA* which was down regulated in young patients with well differentiated tumors, and *NPY* which was up-regulated in old patients with well differentiated tumors. *FOLH1(PSMA)* a male reproductive organ cancer associated gene was up-regulated in young patients with well differentiated tumors, potential target of toxin-based immunotherapy. The other significant findings were *RARRES1* was down regulated in old patients, implicated in retinoid therapy and found to be as a tumor suppressor for multiple cancers such as prostate, breast, gastric, leukemia (25, 26). It has been shown that prostate-specific membrane antigen (PSMA) has potential for the management of prostate cancer chemoprevention by phytochemicals which is emerging as a potential adjunctive approach for the treatment of early carcinogenic processes (27). Further, several other genes such as *LDH-B* described as hypermethylated in prostate cancer; *ID4*, potential tumor suppressor gene in prostate cancer; *ANXA2* indicator of poor prognosis, recurrence, metastasis, high Gleason; *PSGR*, potential serum biomarker of prostate cancer; *ID2*, a p53 independent anti-apoptotic function in prostate cancer cells was found to be down regulated; and *MEIS2*, which act as putative tumor suppressor genes in prostate cancer; NPY, differentially expressed and up-regulated in 60% of “non-aggressive” tumors. *ERG* can fuse with *TMPRSS2* promoter to form an oncogenic fusion gene that is commonly found in human prostate cancer, especially in hormone-refractory prostate cancer. This gene encodes a member of the erythroblast transformation specific (ETS) family of transcriptions factors. All members of this family are key regulators of embryonic development, cell proliferation, differentiation, angiogenesis, inflammation, and apoptosis. *ERG* found to be up regulated in young patients with both well and poorly differentiated tumor and in old only in well differentiated tumor. Interestingly, *ERG* alterations were not found in old men with poorly differentiated tumor.

**Figure 9:**
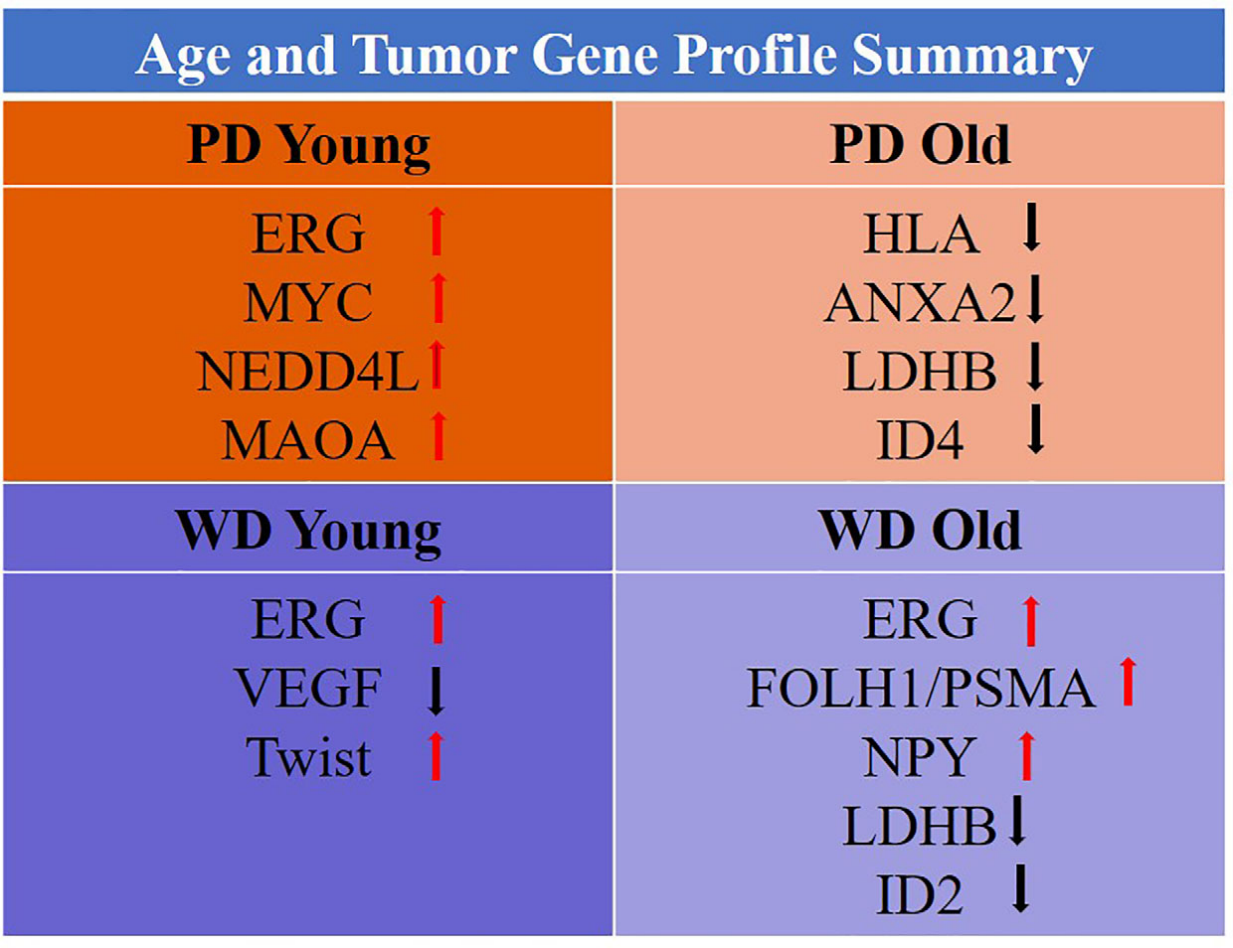
Gene profile of old/young age-associated well/poorly differentiated tumors.

It is worth discussing the study limitations and strengths of the study. The small sample size, low power, in some extent the age difference between young and old (only ~9.9 to ~14.2 years) and lack of biological data currently are some of the limitations of the study. Additional sample sizes and with different ethnic backgrounds specifically African Americans are needed to further extent this study. The strengths of this study are 1) RNA from single malignant and normal epithelial cells from same patient, 2) high stringency of gene selection (T/N > Factor 3), 3) minimized normal cell aging variability, and 4) multiple known prostate cancer genes ID’s which were common among the age groups. Our results are the first in the literature to suggest the existence of strong age disparateness in gene expression among old and young well and poorly differentiated tumors. The unique feature of this study is the robust enrichment of age-associated prostate tumor gene expression signature achieved by subtraction of normal aging signature of prostate epithelial cells of non-familial prostate cancer patients. The differential gene expression levels appear to be very polarized; tumors of young prostate cancer patients bear more oncogenic expressions as compared to old men and old men have more loss of tumor suppressor as when compared to young group. The gene profile of old and young age-associated well and poorly differentiated tumors are summarized in **Figure 9** and age-associated differences in gene expression signatures in poorly differentiated prostate tumors were presented in **Supplementary Figures 3A, B**. Further we have extended our work to confirm and validate these findings in a larger prostate cancer TCGA RNA-seq cohort. The RNA-seq gene expression signature along with clinical data within the age and differentiation threshold were consistent with our discovery cohort. It has been well established that alterations in various molecular genetic mechanisms, including mutations and epigenetic changes followed by perturbations in cell signaling and metabolic pathways are involved in prostate cancer development. Genes, which participate in these pathways, can serve as either diagnostic or prognostic biomarkers. As prostate cancer is a late onset disease and the genetic, ethnic and familial factors being responsible for this occurrence. A very limited information on early-onset of prostate cancer as well as its causes and trends are available. The main focus of this research study to develop only age and tumor differentiation associated gene signature of non-familial prostate cancers in Caucasian men. Further extension of this study is needed in other ethnic populations to develop global age and tumor specific biomarker panel for systemic progression, PSA Recurrence and prostate cancer therapy.

## Conclusions

**Figure 10:**
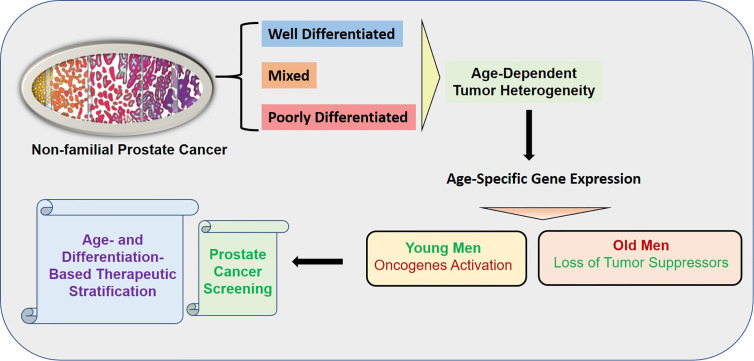
Graphical Abstract: The overall summary of the findings is presented in the graphical abstract. The age-dependent heterogeneity was found to be associated with tumor differentiation. The activation of oncogenes was found in the young patients whereas loss of tumor suppressors was found in old patents. The uniqueness of the study is the robust enrichment of age-associated prostate tumor gene expression signature achieved by subtraction of normal aging signature of prostate epithelial cells of non-familial prostate cancer patients.

Age is a risk factor of cancers and age-associated differences in clinical outcome have been established in prostate cancer. Prostate cancer in young men appears to be composed predominantly of overexpressed genes known to be associated with somatic genomic alterations in prostate cancer. In contrast, prostate cancer of old men appears to have mostly down-regulated gene expressions indicating the loss of protective genes. The age-dependent heterogeneity was found to be associated with tumor differentiation. The overall summary of the findings is presented in the graphical abstract (**Figure 10**).

## Clinical Significance

The difference in prostate cancer of young and old men suggest a distinct biology among these groups. The unique age specific gene expression signature showed oncogenic activation in young and loss of tumor suppressors in old prostate cancer patients, which suggests fundamental differences in tumor development based on aging. This age dependent tumor heterogeneity of non-familial prostate cancer will not only establish prostate cancer screening but also will serve as age-and differentiation based therapeutic stratification of prostate cancer. In young-PD patients *ERG/MYC/NEDD4L/MAOA* oncogene panel was found to be activated whereas in old-PD patients *HLA-A/B*/*ANXA2/LDHB/ID4* tumor suppressor panel was down regulated. In WD-old group *ERG/FOLH1/PSMA/NPY* were up and LDHB/ID2 were down regulated however in WD-young *ERG/Twist* were up and *VEGF* was down regulated (**Figure 9**). These findings suggest some advantage of immunotherapy in old-PD patients and BET bromodomain inhibitors in young-PD, which block prostate cancer cell growth through c-MYC and androgen receptor (AR) suppression can be used in the prostate cancer subset of patients based on the age and tumor type.

## Data Availability Statement

The datasets presented in this study can be found in online repositories. The names of the repository/repositories and accession number(s) can be found below: https://www.ncbi.nlm.nih.gov/, GEO Accession: GSE32448; the recount2 project for 319 cases at https://jhubiostatistics.shinyapps.io/recount/; and https://portal.gdc.cancer.gov/projects/TCGA-PRAD.

## Ethics Statement

The studies involving human participants were reviewed and approved by Walter Reed National Military Medical Center (WRNMMC) and Uniformed Services University of the Health Sciences (USUHS). Written informed consent for participation was not required for this study in accordance with the national legislation and the institutional requirements.

## Author Contributions

SSh, AD, and TA conceptualized the study. SSh and TA contributed to the methodology, SSh and TA provided the software. SSh and TA conduted the formal analysis. SSr, IS, and JC conducted the investigation.AD, JC, IS, DM, and SSr provided the resources. SSh and TA conducted the data curation. SSh and TA wrote and prepared the original draft. HL, SSh, and TA wrote, reviewed, and edited the manuscript. SSh and TA conducted the visualization. DM, AK, NW, and AS conducted the TCGA data analysis and validation. SSh and AD supervised the study. AD, HL, SSr, DM, ATK, NW, and AS revised the manuscript. SSh and TA conducted the project administration. DM and SSr acquired the funding. All authors contributed to the article and approved the submitted version.

## Funding

This work has been supported by funding from Center for Prostate Disease Research, Uniformed Services University for the Health Sciences (HU0001-17-2-2019, HU0001-10-2-0002 and HU001-004-c-1502 to DM, IR, and SSr) from the office of Congressionally Directed Medical Research Programs (CDMRP) of the US Army Medical Research and Materiel Command (USAMRMC) and the Intramural Research Program of the National Cancer Institute, National Institutes of Health.

## Disclaimer

The contents of this publication are the sole responsibility of the author(s) and do not necessarily reflect the views, opinions, or policies of Uniformed Services University of the Health Sciences (USUHS), The Henry M. Jackson Foundation for the Advancement of Military Medicine, Inc., the Department of Defense (DoD), the Departments of the Army, Navy, or Air Force. Mention of trade names, commercial products, or organizations does not imply endorsement by the U.S. Government.

## Conflict of Interest

The authors declare no conflict of interest. The funders had no role in the design of the study; in the collection, analyses, or interpretation of data; in the writing of the manuscript, or in the decision to publish the results.
