# Supplementary material for: Age and Tumor Differentiation-Associated Gene Expression Based Analysis of Non-Familial Prostate Cancers
Source: Front Oncol. 2021 Jan 26;10:584280. doi: 10.3389/fonc.2020.584280 (PMC7870995; doi:10.3389/fonc.2020.584280)
Supplement: Supplementary file 1 [file Table_1.docx]

Supplementary Material

**Supplementary Table 1 (A-D)**: Clinicopathological features of well, poorly and balanced differentiation patients and recurrent PSA group


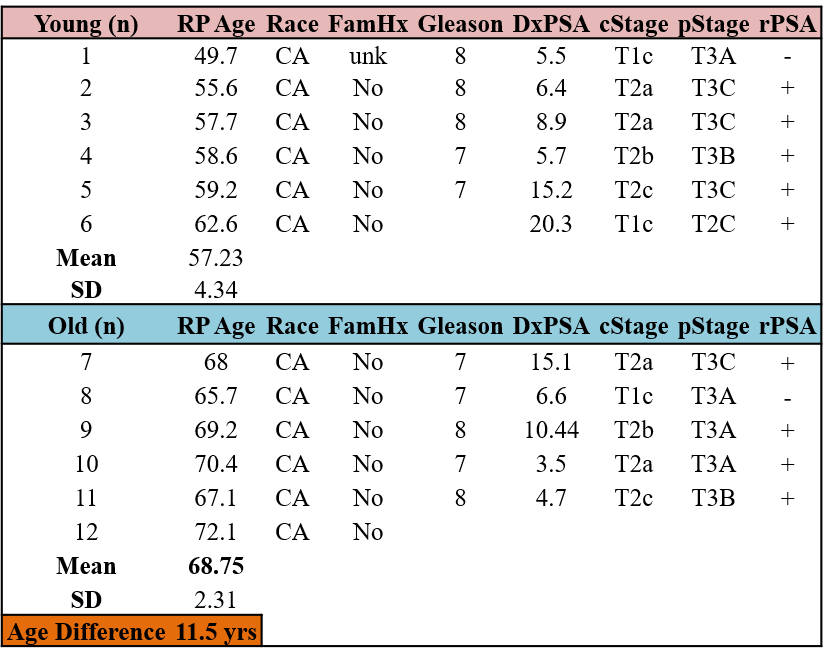
**Suppl. Table 1A: Poorly Differentiated**

**Suppl. Table 1B: Well Differentiated**


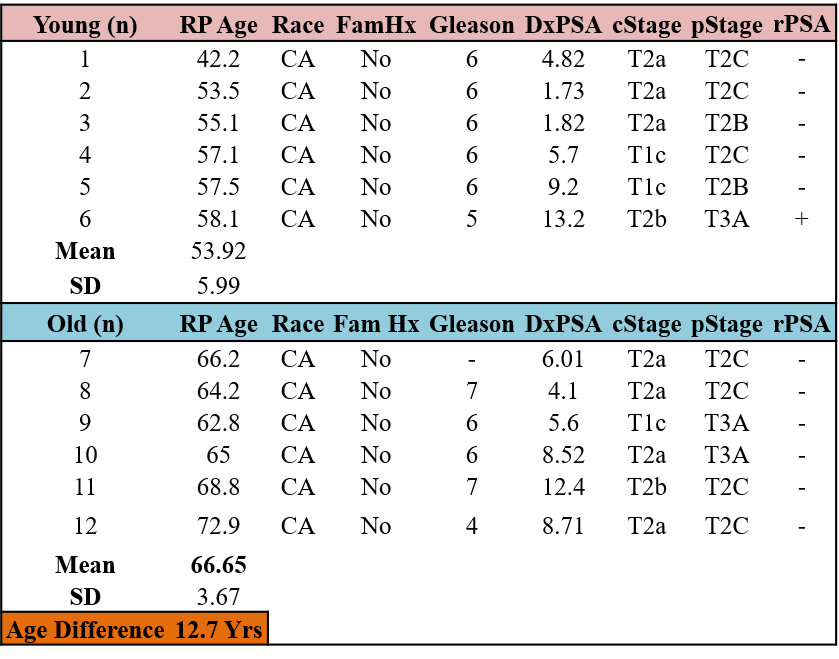


***
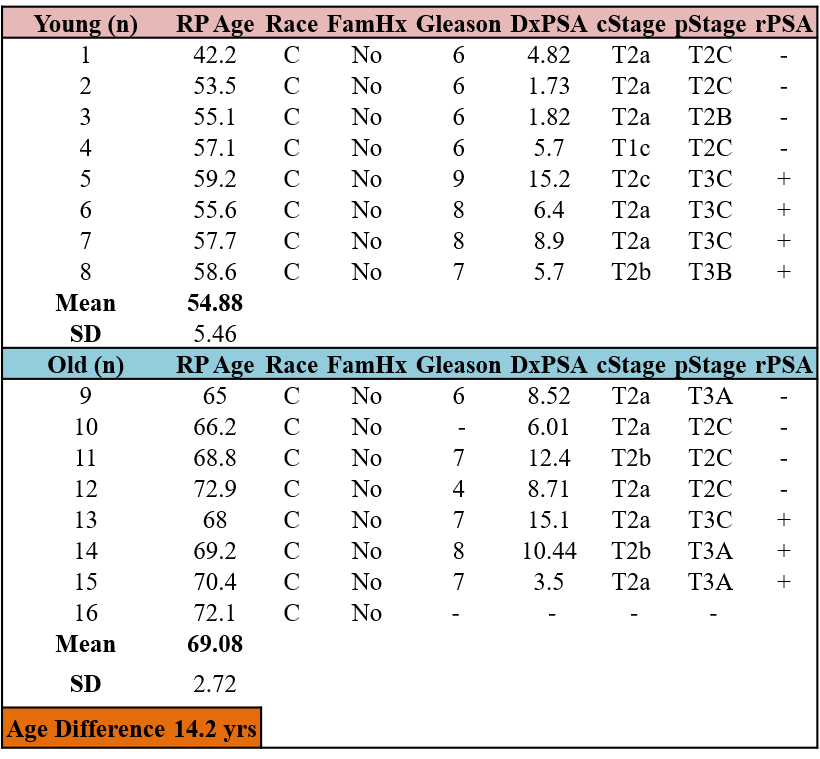
* Suppl. Table 1C: Balanced Differentiated**


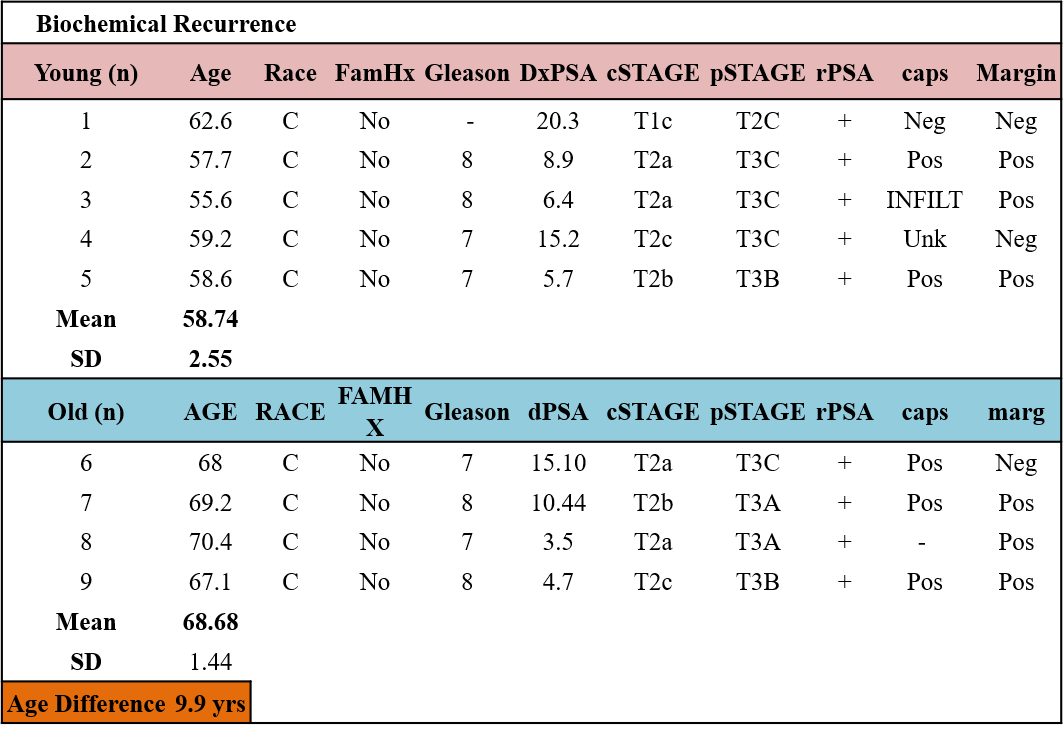
 **Suppl. Table 1D: PSA Recurrence**

**Supplementary Table 2 (A-B)**: Clinicopathological features of well and poorly differentiated in old and young patients from the prostate cancer TCGA cohort

**Suppl. Table 2A: Old Age Well and Poorly Differentiated**

| **Well Differentiated OLD [66-73] (n=6)** | | | | | | | | | |
| --- | --- | --- | --- | --- | --- | --- | --- | --- | --- |
| **Patient ID** | **RP Age** | **Race** | **FamHx** | **Gleason** | **DxPSA** | **cStage** | **pStage** | **rPSA** |  |
| TCGA-CH-5768- | 72 | CA | unk | 6 | unk | -- | T3a | - |  |
| TCGA-EJ-7317- | 71 | CA | unk | 7 | unk | T1c | T2c | - |  |
| TCGA-EJ-7794 | 67 | CA | unk | 7 | unk | T2c | T2c | - |  |
| TCGA-G9-6348 | 68 | CA | unk | 7 | unk | T3a | T2c | - |  |
| TCGA-G9-6365 | 71 | CA | unk | 7 | unk | T2a | T4 | - |  |
| TCGA-J4-A83J- | 68 | CA | unk | 7 | unk | T1c | T2c | - |  |
| **Mean** | **69.50** |  |  |  |  |  |  |  |  |
| SD | 2.07 |  |  |  |  |  |  |  |  |
| **Poorly Differentiated OLD [66-73] (n=6)** | | | | | | | | | |
| **Patient ID** | **RP Age** | **Race** | **FamHx** | **Gleason** | **DxPSA** | **cStage** | **pStage** | **rPSA** |  |
| TCGA-CH-5767 | 66 | CA | unk | 7 | unk | '-- | T2c | - |  |
| TCGA-EJ-7315 | 68 | CA | unk | 7 | unk | T2a | T3a | - |  |
| TCGA-EJ-7330 | 68 | CA | unk | 7 | unk | T1c | T3a | - |  |
| TCGA-EJ-7783 | 70 | CA | unk | 7 | unk | T2c | T3a | + |  |
| TCGA-G9-6333 | 66 | CA | unk | 7 | unk | T1c | T2c | - |  |
| TCGA-G9-6499 | 66 | CA | unk | 9 | unk | T3a | T3a | - |  |
| **Mean** | **67.33** |  |  |  |  |  |  |  |  |
| SD | 1.63 |  |  |  |  |  |  |  |  |

**Suppl. Table 2B: Young Age Well and Poorly Differentiated**

| **Well Differentiated YOUNG [42-58] (n=9)** | | | | | | | | | |
| --- | --- | --- | --- | --- | --- | --- | --- | --- | --- |
| **Patient ID** | **RP Age** | **Race** | **FamHx** | **Gleason** | **DxPSA** | **cStage** | **pStage** | **rPSA** |  |
| TCGA-EJ-7125 | 44 | CA | unk | 7 | unk | T1c | T2c | - |  |
| TCGA-EJ-7785 | 54 | CA | unk | 7 | unk | T2b | T3a | - |  |
| TCGA-EJ-7792 | 53 | CA | unk | 7 | unk | T1c | T2c | - |  |
| TCGA-EJ-7793 | 49 | CA | unk | 7 | unk | T2c | T2c | - |  |
| TCGA-EJ-7797 | 53 | CA | unk | 7 | unk | T1c | T2c | - |  |
| TCGA-G9-6351 | 51 | CA | unk | 7 | unk | T1c | T2c | - |  |
| TCGA-G9-6384 | 53 | CA | unk | 7 | unk | T1c | T3a | - |  |
| TCGA-HC-7738 | 58 | CA | unk | 7 | unk | '-- | T2c | unk |  |
| TCGA-HC-7742 | 58 | CA | unk | 7 | unk | '-- | T3a | - |  |
| **Mean** | **52.56** |  |  |  |  |  |  |  |  |
| SD | 4.33 |  |  |  |  |  |  |  |  |
| **Poorly Differentiated YOUNG [42-58] (n=4)** | | | | | | | | | |
| **Patient ID** | **RP Age** | **Race** | **FamHx** | **Gleason** | **DxPSA** | **cStage** | **pStage** | **rPSA** |  |
| TCGA-CH-5769 | 48 | CA | unk | 9 | unk | '-- | T3b | - |  |
| TCGA-G9-6362 | 57 | CA | unk | 7 | unk | T1c | T3a | - |  |
| TCGA-G9-6496 | 61 | CA | unk | 7 | unk | T1c | T2c | - |  |
| TCGA-HC-8262 | 57 | CA | unk | 8 | unk | '-- | T2c | - |  |
| **Mean** | **55.75** |  |  |  |  |  |  |  |  |
| SD | 5.50 |  |  |  |  |  |  |  |  |


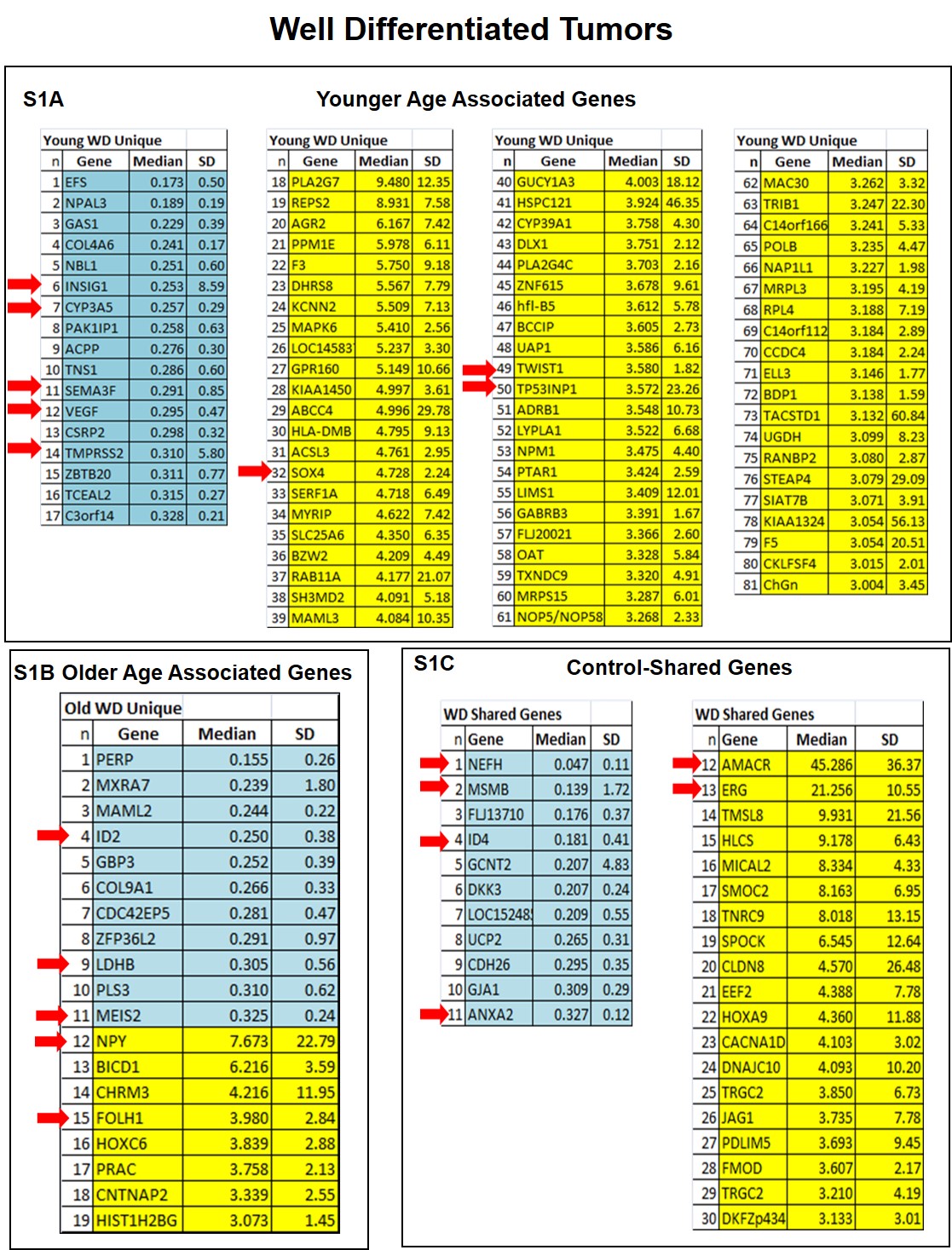


Supplementary Figure 1: Differentially expressed genes in well differentiated tumors of young and old prostate cancer patients.


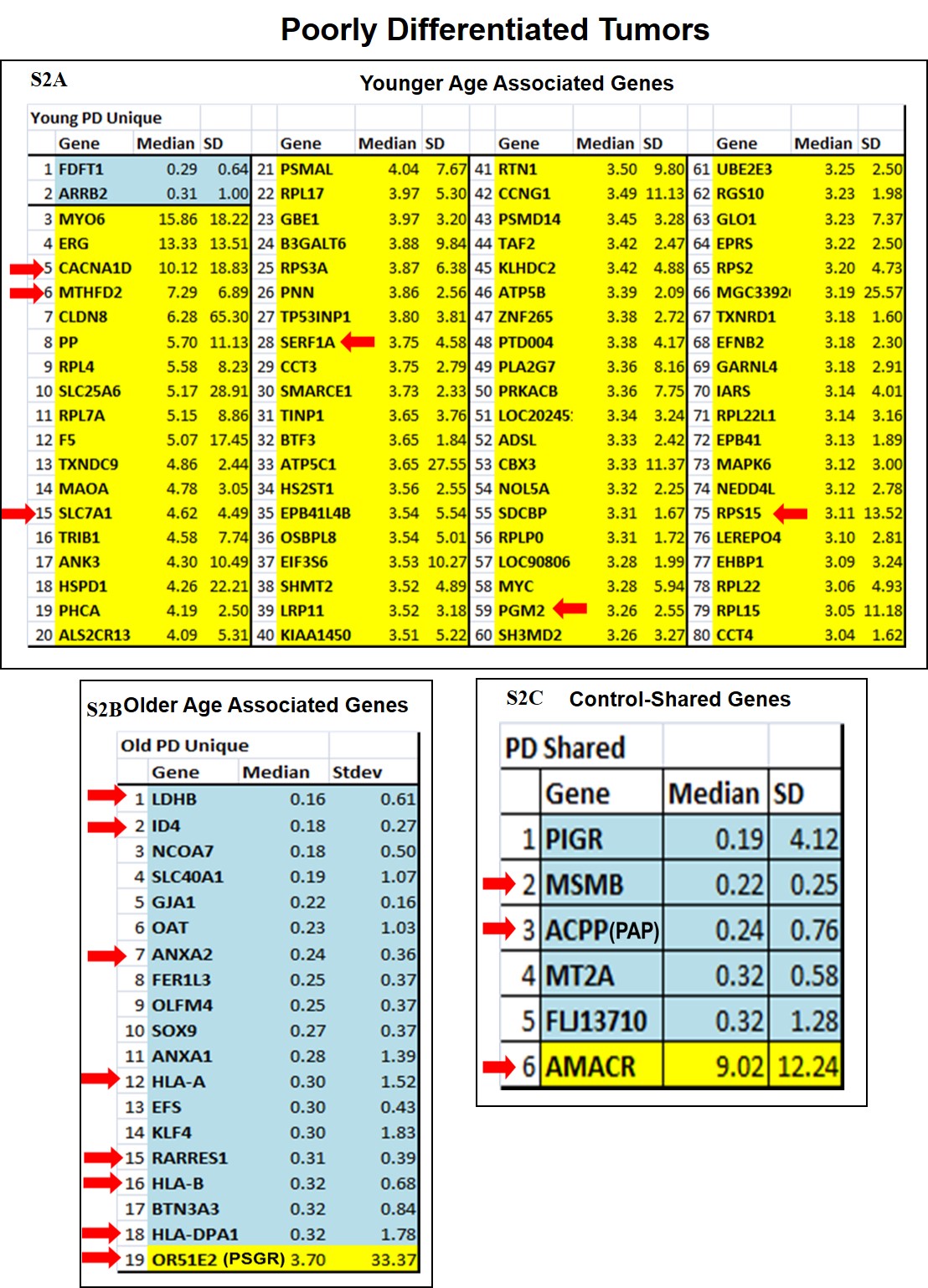


Supplementary Figure 2: Differentially expressed genes in well differentiated tumors of young and old prostate cancer patients.

**
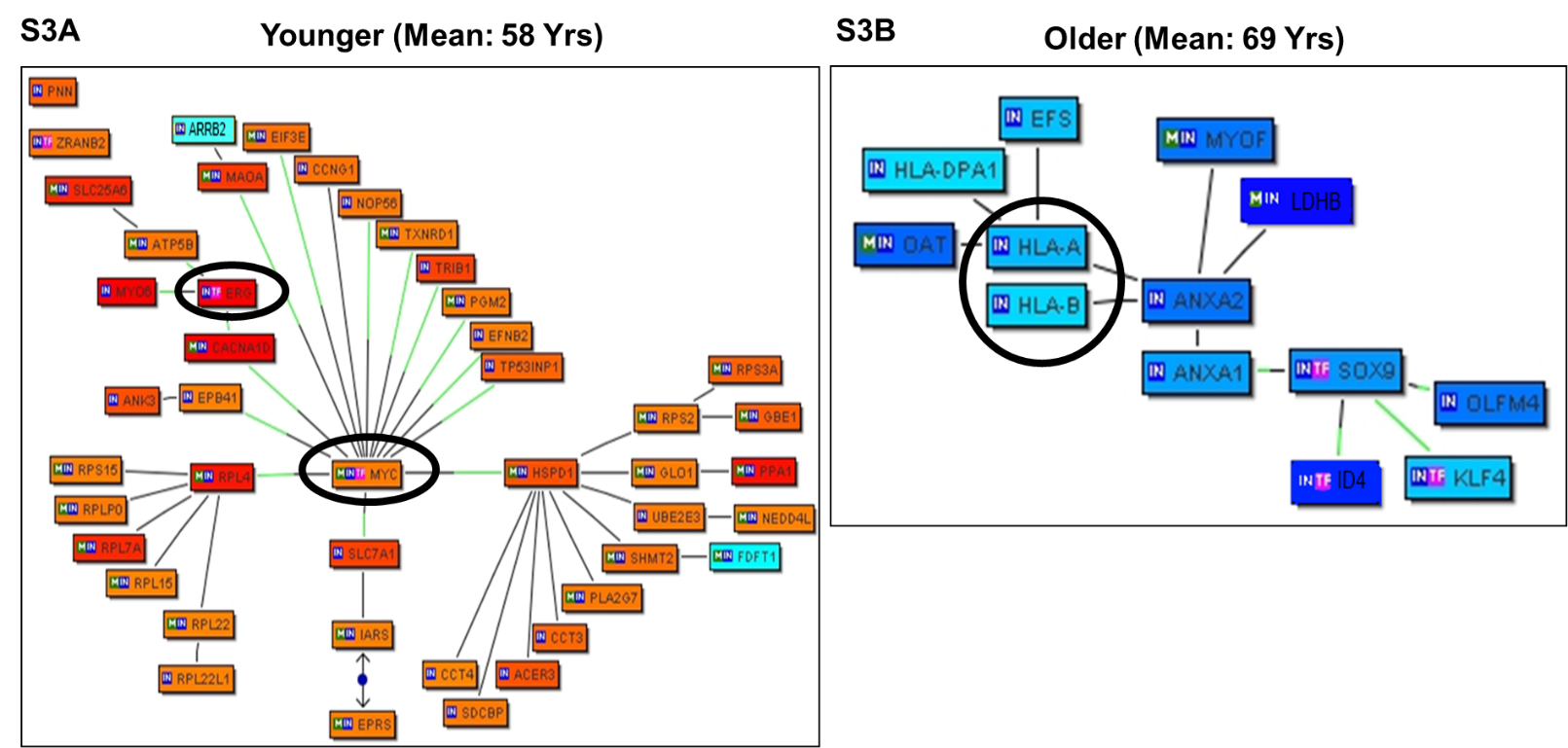
**

**Supplementary Figure 3:** Marked Age-associated Differences in Gene Expression Signatures of Poorly Differentiated Prostate Tumors**. (A)** *MYC/ERG* expression were elevated in younger patients à activation of oncogenes. **(B)** *HLA-A/B* expression were decreased in older patients.
